# Clinical characteristics of immune tolerance after pediatric liver transplantation

**DOI:** 10.1186/s12893-021-01402-0

**Published:** 2022-03-19

**Authors:** Yan Tang, Jingyu Chen, Bailin Chen, Chunbao Guo

**Affiliations:** 1 Department of Pediatric General Surgery, Children’s Hospital of Chongqing Medical University, 136 Zhongshan 2nd Rd., Chongqing, 400014 People’s Republic of China; 2 Department of Ultrasound, Children’s Hospital of Chongqing Medical University, Chongqing, 400014 People’s Republic of China; 3 Ministry of Education Key Laboratory of Child Development and Disorders; National Clinical Research Center for Child Health and Disorders; China International Science and Technology Cooperation Base of Child Development and Critical Disorders; Chongqing Key Laboratory of Pediatrics, Children’s Hospital of Chongqing Medical University, Chongqing, People’s Republic of China

**Keywords:** Immune tolerance, Liver transplantation, Biliary atresia, Immunosuppression withdrawal

## Abstract

**Background:**

Clinical operational tolerance is the ultimate goal for liver transplantation. This study aimed to investigate the clinical characteristics of immune tolerance after pediatric liver transplantation and to identify the possible predictors.

**Methods:**

The clinical data from 37 cases of pediatric patients 2 year later after liver transplantation surgery in the Children’s Hospital of Chongqing Medical University, China, were retrospectively analyzed. According to the status of the current immunosuppressant medications of the patients, they were divided into tolerance (n = 15) and Control (n = 22) groups. The current status regarding prope/operational tolerance was reviewed and screened based on the immunosuppressant medications.

**Results:**

The patients in the tolerance group were younger than that of Controls (p < 0.001). The children in the tolerance group experienced no acute rejection episode and exhibited no obvious abnormalities in the liver function during the continuous follow-up period. The primary disease of the tolerance group were more often diagnosed with biliary atresia (p = 0.011), and received with a living donor liver graft (p = 0.005). There were less glomerular function, diabetes mellitus, arterial hypertension events presented in the tolerance group compared with the control group, indicating low toxicity profile.

**Conclusion:**

In the current study, there were really certain quantity of recipients following liver transplantation attained long term immune tolerance, with low toxicity and satisfied liver graft function. The younger age of the recipient and maternal donor seems to promote long-term clinical immune tolerance. Further work in larger series should be required to describe the overall perspective of tolerance.

## Background

Liver transplantation has been recognized as the most effective and ultimate treatment for irreversible acute or chronic liver disease, with satisfactory results in short- and long-term survival rates. However, similar to cases of the transplantation of many other organs, the overall life expectancy of liver transplant patients is still lower than that of the general population. This is primarily due to the compulsory lifelong use of immunosuppressant(s) to prevent transplant failure. The adverse reactions to these drugs include nephrotoxicity, diabetes, cardiovascular disease, metabolic syndrome, and bone loss. Furthermore, an opportunistic pathogen infection or cancer may potentially occur. The best way to solve these problems is to induce specific tolerance in the liver transplant recipient, thus achieving the long-term survival of the allograft without long-term immunosuppression. The immunosuppression (IS)-free state represents the ideal goal of the posttransplantation immunosuppression management. The operational tolerance is described as an IS-free state with allograft acceptance without a specific protocol to induce tolerance. For some transplanted patients, a very low-dose immunosuppressive agent maintenance, may guarantee sufficient graft acceptance against rejection, termed as prope (‘almost’ in Latin) tolerance.

Transplantation tolerance has several typical features. For example, the allograft can maintain normal function and morphology without using an immunosuppressant, thereby prolonging the allograft survival; an in vitro test reveals no or only a weak donor-specific reaction, and there is spontaneous acceptance of an allograft from a second-party donor with the rejection of an allograft from a third party [1]. Unlike acute or chronic rejection, our understanding of clinical immune tolerance is lacking. In some rare cases, such as when patients are non-compliant with immunosuppressive therapy, in cases of clinical trials in which the immunosuppressant is intentionally withdrawn, or in patients who have severe clinical considerations (lymphoproliferative disorder or life-threatening infections), the phenomenon of scattered tolerance can be observed [2–5]. This phenomenon of clinical tolerance has gained increasing attention in the past 20 years [6–8]. In addition, the results of a multi-center study indicated that immune tolerance was more common in children than in adults.

In clinical practice, we have also observed the tolerance of patients after withdrawing immunosuppressive drugs. To investigate the clinical characteristics, immunological, and toxicity status affecting postoperative immune tolerance after pediatric liver transplantation, the clinical data from 37 cases of pediatric patients who underwent liver transplantation surgery in the Children’s Hospital of Chongqing Medical University, China, were retrospectively analyzed.

## Methods

### Subjects

This retrospective analysis was conducted following expedited approval from the Research Ethics Committee of Chongqing Medical University under the protection of personal information. The electronic clinical record of the pediatric (< 18 years of age) recipients of a liver transplant from July 1, 2005, to May 30, 2018 were reviewed. The patients underwent the liver transplantation surgery and routinely follow-up in our hospital at least 2 years after the transplantation, and the examination and medication data recorded at each follow-up meet the inclusion criteria. Exclusion criteria were biliary complications involvement during the follow up period, death within 2 years after the transplantation and the cases with missing follow-up data, like the pathological results.

### Medication

#### Immunosuppression protocol

Postoperative immunosuppression was initially tacrolimus (Tac) or cyclosporine-based (relevant with patient’s age and transplant era), in combination with steroids. The postoperative doses were adjusted on the basis of recommended trough blood concentrations at different stages. Tac was converted to CyA or rapamycin (Rap) when Tac-related adverse events occurred. Steroids and MMF were eventually discontinued after 6 months. There were always many patients with poor compliance with the prescription, for the patients with abnormal liver function test, the IS medication should be adjusted or resumed. For the patients exhibited no obvious abnormalities in the liver function during the continuous follow-up period after stopping the medication, the IS medication was withdraw of their own accord thereafter. Here we intend to investigate the state of allograft acceptance following immunosuppressive drugs withdrawing, no matter whether they were compliant with the prescription or not.

According to the immunosuppressant medications and the evaluation for their liver function during followup visit, the included children were categorized into two groups: (1) tolerance group, including operational and prope tolerance, which was defined that if they did not have acute or chronic rejection for at least 1 year off immunosuppressive medications or monotherapy with trough blood concentrations under the lower limit of the therapeutic range, combined with liver tests, including liver function and histological evaluation. We have confirmed the ISP levels when the patients were taken the very low dose or no immunosuppressive agent for at least 1 year. During this time period, the medication was continued and clear. Here the poor compliance occurred at least 1 year before the confirmation of the prope tolerance and operational tolerance.

(2) Control group, any time with the abnormal liver evaluation an increase in aspartate aminotransferase (AST) or alanine aminotransferase (ALT) levels to greater than twice the upper baseline value without any other cause and without any postoperative complications a year later the liver transplantation surgery.

### Data collection and outcome

The possible influencing factors in the medical records were screened based on the clinical data, which included the following: the demographic data, surgical, and laboratory variables, the IS therapy strategy, the IS-related toxicity effects, the blood type of the transplant recipient, the primary disease, the gender and age at transplantation of the donor, the last liver allograft histology and the liver function during the postoperative follow-up period. These liver function indicators were obtained based on the average of the data from a previous follow-up.

### Statistical analysis

First, a univariate analysis was carried out for all the possible influencing factors, and the differences among the two groups were compared to initially identify the relevant factors. The measurement data used for this purpose were tested for normality according to the Kolmogorov–Smirnov method, and the normally distributed measurement data were then further tested for homogeneity of variance. The data exhibiting a normal distribution and homogeneity of variance were compared with an analysis of variance for multiple-sample means, while the rank sum test of multiple independent samples was performed for the non-normally distributed data. The countable data were compared using the χ^2^ test of the R × C table, and Fisher’s exact probability was calculated for those not satisfying the criteria of the χ^2^ test. The categorical data were compared mainly using the rank sum test of multiple independent samples. The SPSS19.0 (IBM, Armonk, NY, USA) statistical software was used for the data processing. According to the significance level α = 0.05, differences with p < 0.05 were considered statistically significant.

## Results

During the period of research time, there were 137 cases of pediatric liver transplantation recipients (median follow-up: 2.6 year) in our hospital were reviewed. Among the whole cohort following liver transplantation, there were altogether 42 patients not compliant with the prescription, 3 patients with unobtainable data and 2 patients initially managed in other centers, which were eligible for analysis. Overall, 37 were finally included in the current research. The primary diseases of the pediatric patients receiving liver transplantation included biliary atresia (n = 26, 70.3%), followed with glycogen storage disease (n = 4, 10.8%), Wilson’s disease (n = 2, 5.4%), and unexplained hepatic cirrhosis (n = 6, 16.2%) (Table 1). There were 15 cases categorized into tolerance group with 4 patients complete withdraw of the immunosuppression medication, the remaining LT recipients constituted the Control group (n = 22).

**Table 1:** Diagnostic evaluation for the patients

Parameters	Tolerance (n = 15)	Controls (n = 22)	p value
Liver function tests			
TB (2.0–20), umol/L, Median (range)	13.75 (8.10–22.30)	16.81 (9.30–33.50)	0.13
DB (0–4), umol/L, Median (range)	3.1 (0–5.2)	4.5 (0–8.9)	0.38
ALT (0–50), U/L, Median (range)	36.5 (13–91)	53.2 (11–324)	0.016
AST (0–50), U/L, Median (range)	31 (17–78)	59 (23–416)	0.003
rGT (0–50), U/L, Median (range)	21.5 (5–142)	29.3 (6–286)	0.13
Histological findings			
Normal liver biopsy	11	10	0.089
Borderline IS	0	6	0.032
Non-specific hepatitis	4	5	0.54
Fibrosis	2	8	0.15

Table 1 compares the liver function tests of the last tests and liver biopsy in both groups. The Kruskal–Wallis test was performed for comparisons of the levels of ALT, AST, GGT, TBIL, DBIL and the results showed statistically significant differences in the ALT and AST levels (p = 0.016 and p = 0.003, respectively). The liver function tests in the tolerance group presented not exceeding more than the upper limits, which was in accordance with the prope/operational tolerance definition. No statistically significant difference in the TBIL, DBIL levels among the two groups was found using the SNK-q test (Table 1). The pathological findings in terms of the histological injury features were also indicated in Table 1 and the results showed that about 73.3% (11/15) of patients in the tolerance group presented with normal biopsy compared with about 45.5% (10/22) of the biopsy in the Control group (p = 0.089).

Comparable demographic data of two groups are summarized in Table 2. In this study, Fisher’s exact test was performed for the countable data including gender, blood type, transplant mode and primary disease. Pretransplant diagnosis of biliary atresia was more common in the tolerance group then in the Control group (p = 0.011). Further pairwise comparisons between the groups revealed a statistically significant difference in the age of the recipients at transplant between the tolerance group and the Control group (Table 2). The patients in the tolerance group were younger than that of control (p < 0.001). The primary disease were significantly more biliary atresia for the tolerance group compared with control (p = 0.011), accordingly transplanted with more living related donor liver graft (p = 0.005).

**Table 2:** Clinical parameters of the patients for comparison in the two groups

	Tolerance(n = 15)	Controls (n = 22)	p value
Age of the recipient at transplant, Mon, Median (range)	6.2 (2.8–10.3)	14.5 (5.7–156.6)	< 0.001
Primary disease			
Biliary atresia	14	11	0.011
Glycogen storage disease	0	4	0.13
Wilson’s disease	0	2	0.51
Unexplained hepatic cirrhosis	1	5	0.37
Transplant mode			
LDLT	15	13	0.005
DCD	0	9	
Donor’s gender			
M	2	10	0.073
F	13	12	
Recipient’s gender			
M	7	9	0.75
F	8	13	
Blood matching			
Identical	6	10	0.51
Compatible	9	12	0.51
Incompatible	0	0	–
Time post-LT (m)	48.6 (23.5–79.2)	35.3 (15.2–112.6)	0.26

The side effects, which should be related with the IS were detailed in Table 3. there were less diabetes mellitus, arterial hypertension, glomerular function events presented in the tolerance group, without diabetes mellitus or hypertension presented, and there were a few side effects, related with the IS in the Control group. There were no significantly difference observed between the two groups.

**Table 3:** Long term side effects for the patients with different medications

Factor	Tolerance (n = 15)	Controls (n = 22)	p value
Abnormal renal function, n (%)	0	2	0.51
Hypertension	0	0	–
Diabetes mellitus	0	1	1.00
PTLD	1	2	1.00

## Discussion

The clinical tolerance refers to a case in which no specific destructive immune response against the graft is detected without the use of any immunosuppressive drug [9–12]. Until now no consensus has been reached for parameter to indicate the immunosuppressive weaning or how long the maintenance of stable function must be for a definition of tolerance. In addition, there are many other definitions of tolerance, like prope/operational tolerance [13, 14]. In the present research, the state of prope or operational tolerance have been presented in a significant proportion of LT recipients, indicating a validated and achievable operation for pediatric patients.

A research team in Pittsburgh described immune tolerance for the first time in 1990 [15]. Later, the hospital of Tokyo University reported that 88 out of 581 cases of pediatric related LDLT recipients (15%) displayed immune tolerance [16]. In 2012, the University of California, San Francisco conducted a prospective, multicenter, and open-label pilot study of 20 cases of pediatric LDLT recipients with stable disease, and 60% of the pediatric LDLT recipients maintained normal function and stable morphology of the graft after the withdrawal of immunosuppression for at least 1 year, thus achieving immune tolerance [17]. In addition, the postoperative termination of immunosuppression after a transplant has also randomly been reported in many countries. Currently, immune tolerance are described as operational tolerance and prope tolerance. Interestingly, operational tolerance was almost always induced following PTLD, whereas it is not applicable for prope tolerance. Although the benefit of them were minimal IS-related toxicity, we still did not know whether the immune mechanisms underlying the prope tolerance are identical to that of operational tolerance.

The medications are troublesome and inconvenient following the liver transplantation in children and adolescents and the non-adherence for the IS management is the leading reason account for the morbidity in this population. In this study, the four pediatric patients withdrew the medication of their own accord without following the doctor’s advice. Fortunately, the continuous follow-up after the withdrawal revealed no abnormality in various indicators, suggesting that the graft function was normal with no rejection or serious adverse events. Accordingly, we believe that these four patients achieved the clinical criteria of operational tolerance and that a state of tolerance had been established. The status of prope tolerance should combine satisfied graft function and low immunosuppressive midication toxicity. There were 11 patients taking only a single immunosuppressant with trough blood concentrations under the therapeutic limit, while maintaining normal graft function, accordingly, these patients presented with low toxicity profile. These patients should be better compliance with the immunosuppressive strategy. This low toxicity profile should be related with an long term advantage in terms of de novo malignancies following the liver transplantation. There are evidence that an incidence risk for cancer increases on the cohort of immunosuppressive treatment following LT [18].

Although the number of patients included in this study was small, especially the number of patients in the tolerence group, a few factors that may be associated with immune tolerance were still found. It is interesting to note that a statistical analysis was performed of the clinical data from 37 cases of pediatric patients who underwent a liver transplantation, and the results revealed differences in the age of the recipients at transplant. Accordingly, we can conclude that the younger age of the recipient at transplantation may be a potential factor influencing the generation of clinical immune tolerance in pediatric liver transplantation. This may be because the immune system gradually matures with increasing age. Between the tolerance group and the Control group, the levels of ALT and AST were all significantly different. As important indicators of liver function, ALT and AST are affected by many factors, such as the function of the liver allograft itself, drugs, and complications. These enzymes should be given more attention in the future in the follow-up of pediatric patients [4, 5].

It is interesting to note that the primary diseases of the pediatric patients in the tolerance group were almost all biliary atresia (14/15, p = 0.011), and the donors were mostly female. Additionally, most cases of liver transplantation were maternal LDLT. Although the analysis showed that these factors were not significantly different among the groups, this result may be because of the small sample size. Further studies for operationally tolerant are needed in terms of the donor gender and recipient features.

We here found that in selected patients after transplantation with manageable risk and acceptable safety minimization of IS with cautious could be conducted to reach operational tolerant. In a future study, a multi-center large-scale data analysis should be performed to further explore the features of immune tolerance. We are currently conduct the prospective study to determine the factors influencing the development of operational tolerant posttransplantation.

## Conclusion

This study showed that there were really tolerance attained in the pediatric LT recipients, with excellent liver graft function. The age of the recipient at transplant may be a potential factor affecting the generation of clinical immune tolerance in pediatric liver transplantation. Obviously, it will be essential to differentiate the cellular and molecular mechanism between the prope and the operational tolerant, which require further work in a larger series.

## Data Availability

The dataset analyzed during the current study is available from the corresponding author on reasonable request.

## Abbreviations

ALP: Alkaline phosphatase
ALT: Alanine aminotransferase
AST: Aspartate aminotransferase
DBIL: Direct bilirubin
DCD: Donor after cardiac death
GGT: λ-Glutamyl transferase
LDH: Lactate dehydrogenase
LDLT: Living donor liver transplantation
TBIL: Total bilirubin
